# Computational and experimental elucidation of the boosted stability and antibacterial activity of ZIF-67 upon optimized encapsulation with polyoxometalates

**DOI:** 10.1038/s41598-022-20392-4

**Published:** 2022-09-26

**Authors:** Aya M. Mohamed, Walaa A. Abbas, Ghada E. Khedr, Wessam Abass, Nageh K. Allam

**Affiliations:** 1 Energy Materials Laboratory (EML), School of Sciences and Engineering, The American University in Cairo, New Cairo, 11835 Egypt; 2 Department of Chemistry, Faculty of Science, Cairo University, Cairo, 12613 Egypt; 3 Department of Evaluation and Analysis, Egyptian Petroleum Research Institute, Cairo, 11727 Egypt; 4 Sustainable Development Program, School of Sciences and Engineering, The American University in Cairo, New Cairo, 11835 Egypt

**Keywords:** Materials chemistry, Chemical synthesis, Biomaterials, Nanoscale materials, Nanoscale materials

## Abstract

Water microbial purification is one of the hottest topics that threats human morbidity and mortality. It is indispensable to purify water using antimicrobial agents combined with several technologies and systems. Herein, we introduce a class of nanosized metal organic framework; Zeolitic imidazolate framework (ZIF-67) cages encapsulated with polyoxometalates synthesized via facile one-step co-precipitation method. We employed two types of polyoxometalates bioactive agents; phosphotungstic acid (PTA) and phosphomolybdic acid (PMA) that act as novel antibacterial purification agents. Several characterization techniques were utilized to investigate the morphological, structural, chemical, and physical properties such as FESEM, EDS, FTIR, XRD, and N_2_ adsorption/desorption isotherms techniques. The antibacterial assessment was evaluated using colony forming unit (CFU) against both *Escherichia coli* and *Staphylococcus aureus* as models of Gram-negative and Gram-positive bacteria, respectively. The PTA@ZIF-67 showed higher microbial inhibition against both Gram-positive and Gram-negative bacteria by 98.8% and 84.6%, respectively. Furthermore, computational modeling using density functional theory was conducted to evaluate the antibacterial efficacy of PTA when compared to PMA. The computational and experimental findings demonstrate that the fabricated POM@ZIF-67 materials exhibited outstanding bactericidal effect against both Gram-negative and Gram-positive bacteria and effectively purify contaminated water.

## Introduction

There has been recently a challenge of shortage in water resources that adversely affect the environment and population. One of the promising solutions to overcome this pressing issue is to disinfect water from microbes^1^. Water microbial purification plays a vital role in infectious disease prevention and microbial inhibition and eradication^1,2^. Bacterial infection threatens not only the environment but also the peoples’ health and this is due to the highly bacterial resistance to disinfectant agents and their ability to make mutations^3,4^. Thus, there is a dire need to eradicate infectious microbes for better quality of water, health care sectors and several industries^2,5^. In addition, to stop the incidence of increasing bacterial infection, novel antibacterial disinfectants agents are needed to limit their spread. The recent use of antimicrobial materials has gained much attention in the field of water microbial purification because of their broad spectrum of antibacterial properties against both types of Gram-positive and negative bacteria; and appropriate properties in the nanoscale range^6^.

Metal–organic frameworks (MOFs) are hybrid organic–inorganic porous crystalline materials which composed of metal nodes connected by organic linkers through coordination bonds^7^. MOFs demonstrates exceptional properties by virtue of their ultrahigh specific surface area, low density, ease of fabrication, high porosity and tunable particle size, morphologies and structures^8^. The outstanding nature of MOF materials and their high surface area to volume ration have been demonstrated due to the microspores within their surfaces and the existence of the functional groups in the organic linkers^9^. Furthermore, the faradic redox metal centers, which are homogenously incorporated within the carbon matrix, enrich the surface with abundant active sites that plays a crucial role in eradicating the bacteria^10^. Moreover, it was remarked that nanoscale MOFs displays an enhanced antibacterial and antiviral activity due to their alternative antimicrobial mechanisms and modes of action resulting in better elimination of bacteria^11,12^. Interestingly, it has been demonstrated that transition metal-based MOFs have a potential role in rupturing the bacterial liquid membranes^13^. This is attributed to the release of transition metal ions such as Co^2+^, Cu^2+^, Zn^2+^, and Mn^2+^, which cause liquid membrane leakage^14^. All of these merits supported MOFs as promising candidates for sensing, biomedicine, drug delivery, antimicrobial, antibacterial and water treatment applications.

Zeolitic imidazolate frameworks (ZIFs) are a sub-class of MOFs that are topological isomorphic with zeolites and exhibits excellent chemical and thermal stability, in particular ZIF-67^15^. The ZIF-67 displays a uniform sodalite structure where each Co^2+^ is tetrahedrally coordinated with four 2-methyle imidazole linkers and each 2-methyle imidazole acts as bridge between two Co^2+^ ^16^. Moreover, it was proved that ZIF-67 exhibits a remarkable antibacterial activity through the controlled release of Co^2+^ which can eliminate the bacteria by destroying their membranes^13,17^. Additionally, ZIF-67 displays a cage-like structure that has the capacity to host other bioactive species within its cavities which results in boosting their antibacterial activity.

Polyoxometalates (POMs) are a class of anionic clusters of transition metal oxides that have unique redox properties. POMs consist of an array of corner- and edge-sharing pseudo octahedrally coordinated units of (typically Mo-, W- and V-oxides) which offer several chemical transformations. They demonstrated outstanding biological activity such as antitumor, antidiabetic, and antiviral and antimicrobial activities. This is attributed to their negative charge, strong acidity, and geometry. Keggin type POMs such as phosphotungstic acid (PTA) and phosphomolybdic acid (PMA) showed promising antibacterial activity against both Gram-negative and Gram-positive bacteria. They act as ‘electron sponge’ owing to their large electron uptake number^11,18–20^. Although POMs lack stability and suffers from dissolution in aqueous electrolytes, incorporating them into MOF materials prevents their dissolution and enhances their stability in several applications, particularly in water microbial purification. Furthermore, their integration with ZIF-67 improves the antibacterial activity of the whole composite material. According to Gumerova et al*.,* POMs antibacterial activity is primarily relying on the composition, size and structure; and in other classes, charge. Additionally, they found that polyoxotungstate exhibited the highest antibacterial activity among other experimented POMs against Gram-negative bacteria *M. catarrhalis* using minimal inhibitory concentration (MIC) test^11^.

We employed two models of bacteria, *Escherichia coli* and *Staphylococcus aureus* as Gram-negative and Gram-positive bacteria, respectively. *Escherichia coli* (*E. coli*) is causing afflictions ranging from inflammations and peritonitis to food poisoning and urinary infections. *Staphylococcus aureus* (*S. aureus*) is the most pathogenic and infectious bacterium that requires hospital-acquired settings. *S. aureus* is a model of a pathogenic microorganism^21^. Basically, the mechanism of water microbial disinfection of metal oxides and MOF depends on the hydroxylation reactions. Hydroxyl and free radicals penetrate the bacterial cell wall and bacterial membrane which act as a strong oxidizing agent for bacterial disinfection in the infected water. Additionally, these free radicals cause adversely effects on bacterial medium leading to serious biochemical and molecular transformations^22^. Moreover, Density functional theory (DFT) calculations is a powerful tool for predicting properties of materials and interpreting data. Nowadays DFT has a great importance in different aspects due to its ability to give fast and credible answers for different materials issues like reaction mechanism^23^, energy barriers^24–26^, optics^27^, drug design^28^.

Herein, we report on the synthesis of ZIF-67 encapsulated Keggin type POMs nanoparticulates in the powder form through a facile one step co-precipitation reaction and demonstrating their antibacterial effect on water bacterial purification using colony forming unit method. The designed composite material (PTA@ZIF-67) demonstrated the highest antibacterial efficiency compared to both the bare MOF and PMA@ZIF-67. Furthermore, the Computational modeling using DFT calculations was conducted to explain and support the experimental findings.

## Experimental section

### Materials

Co(NO_3_)_2_.6H_2_O (cobalt nitrate hexahydrate)—2-methylimidazole (2-MeIM)—phosphomolybdic acid H_3_PMo_12_O_40_ (PMA)—phosphotungstic acid H_3_PW_12_O_40_ (PTA). Antibacterial tests using S. *Aureus* and E. *coli* bacterial strains conducted in Chemistry department, Faculty of Sciences at Cairo University.

### Synthesis of ZIF-67

Facile precipitation method was used to prepare ZIF-67 MOF structure. 0.77 mmol of Co(NO_3_)_2_.6H_2_O (Cobalt nitrate hexahydrate) was dissolved in 5 ml DI H_2_O to form the first solution. Then, 33.5 mmol of 2-methylimidazole (2-MeIM) was further added to 20 ml Methanol to form the second solution. Afterwards, the second solution was added gradually to the first solution. After that, the subsequent mixture was stirred for 2 h. Finally, the purple precipitate has been separated using the centrifugation method and it washed with ethanol several times to discard any unreacted residues. Eventually, the as-prepared (ZIF-67) sample was dried at 60 °C overnight.

### Synthesis of POM@ZIF-67

The POM@ZIF-67 composites were prepared by dissolving 0.77 mmol of Co(NO_3_)_2_.6H_2_O (cobalt nitrate hexahydrate) and 0.05 g of the heteropoly acid (PTA or PMA) in 5 ml DI H_2_O to form solution I. Then, 33.5 mmol of (2-MeIM) was dissolved in 20 ml methanol to form solution II. After that, solution II was gradually added to solution I. The final mixture was stirred for 2 h at room temperature. The next step was to separate the purple precipitate via centrifugation. Finally, the sample was washed with ethanol to ensure the removal of any unreacted residues and it was dried at 60 °C overnight.

### Bacterial counting test/colony forming unit (CFU)

*Staphylococcus* aureus (*S. aureus*) and *Escherichia* coli (*E. coli*) were used to evaluate the antibacterial activity of the ZIF-67 encapsulated POMs MOF nanoparticulates using colony forming unit method. To test the antibacterial ratio of the samples, *S. aureus* and *E. coli* suspension (McFarland standard 0.5) was prepared and incubated in Mueller–Hinton broth medium. The concentration of the sample was 1 mg/mL. Then, 200 μL of the prepared bacterial suspension was added into the plate containing the tested samples and control sample (DMSO), the concentration of the sample was 1 mg/mL. Next, the plate incubated at 37 °C for 24 h. After that, 20 μL bacterial solution was cultured on the surface of dried nutrient agar plates. The plates were incubated at 37 °C for 24 h. The bacterial colony on the plates was observed by a digital camera, and the number of colonies was counted. The antibacterial efficacy was calculated as follows:

$$\mathrm{Antibacterial\, ratio}({\%})=\frac{\left(\mathrm{number\, of\, CFUs\, in\, control\, group }-\mathrm{ number\, of\, CFUs\, in\, experimental\, group}\right)\times 100}{\mathrm{number\,}\boldsymbol{ }\mathrm{of\, CFUs\, in\, control\, group}}$$

### Materials characterization

The surface morphology of the fabricated composite materials was characterized using A Zeiss Ultra 60 field emission scanning electron microscope (FESEM). The crystal structure was investigated using an X-ray diffractometer on an X'Pert PRO MRD with a Cu kα radiation (λ = 0.15406 nm). FTIR analysis were executed on an ATI Unicam (Mattson 936) bench top spectrometer using pressed KBr pellets in the range of 400–4000 cm^−1^. For surface analysis, the N_2_ adsorption/desorption isotherms were tested at −196 °C using Microtrace BELSORP surface area and pore size distribution analyzer. Before the test begins, the samples were degassed under vacuum at 150 °C overnight. The BET model was used to estimate the specific surface area (m^2^ g^−1^). Furthermore, the BJH method was used to evaluate the pore size distribution.

### Computational details

The calculations were done by using the standard Cambridge Serial Total Energy Package (CASTEP) as implemented in Materials Studio version 2017^29^. All of the spin polarization calculations were performed using the generalized gradient approximation of Perdew, Burke and Ernzerhor (GGA-PBE) by Broyden-Fletcher-Goldfarb-Shanno (BFGS) algorithm with ultrasoft pseudopotential. For better accuracy of bandgap calculations, the van der Waals corrections U were involved (U^d^ = 8 eV) for d states of metal atom and (U^p^ = 6 eV) for O 2p states due to DFT self-interaction error. The cutoff energy was set to 380 eV with a convergence criterion of 5 × 10^–6^. The maximum force was less than 0.01 eV/Å and the maximum stress was 0.02 GPa. The Monkhorst–pack k-points sampling were set to a 1 × 1x1 grid. To optimize structures each of PTA and PMA molecule was in a box with dimensions of 20*20*20 Å. All the atoms were relaxed.

## Results and discussion

**Figure 1:**
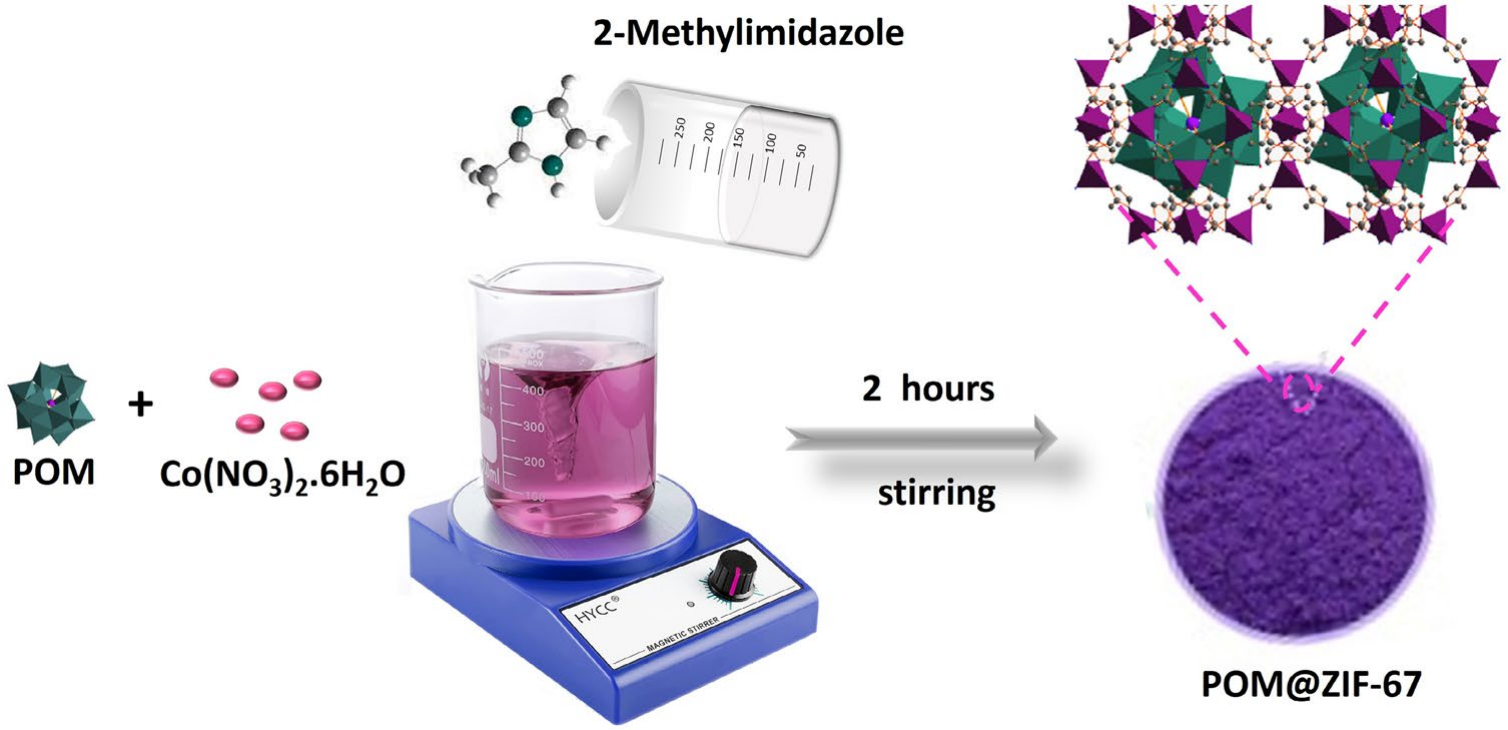
Stepwise Synthesis of POM@ZIF-67.

**Figure 2:**
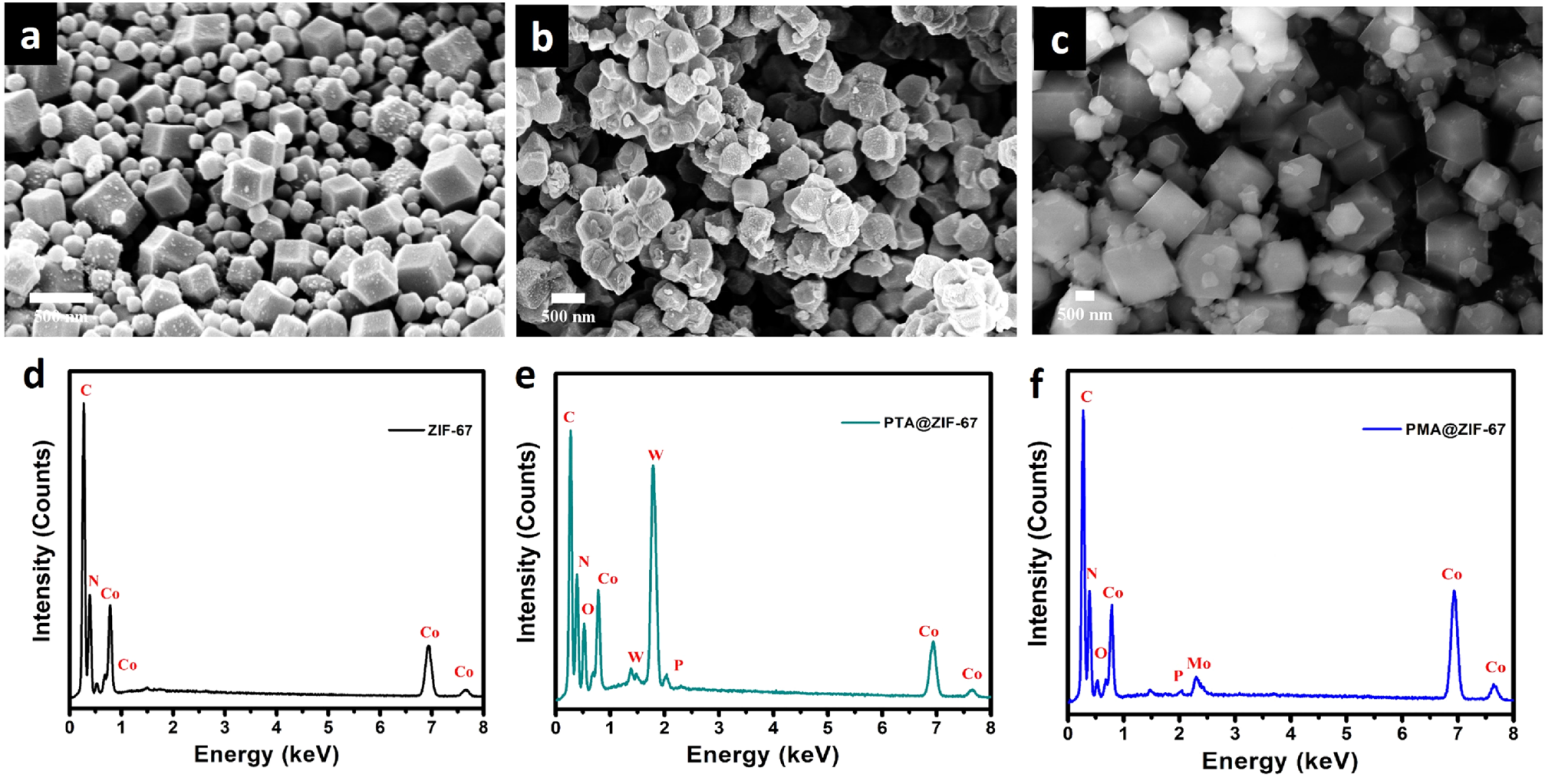
FESEM top view images of the as-synthesized (**a**) pure MOF, (**b**) PTA@ZIF-67, (**c**) PMA@ZIF-67 (**d**) the corresponding EDS spectra of pure MOF, (**e**) PTA@ZIF-67, and (**f**) PMA@ZIF-67.

POM@ZIF-67 hybrids were fabricated through a facile one step coprecipitation reaction at room temperature, Fig. 1. The POM particles were encapsulated within the self-assembled ZIF-67 cages preventing their dissolution. Figure 2a–c displays top-view images of the prepared MOF materials. As depicted from Fig. 2a, it is obvious that the bare ZIF-67 sample displays a uniform polyhedron morphology with an average size of ≈300 nm. Alternatively, the POM encapsulated ZIF-67 samples demonstrated a less uniform structure, Fig. 2b,c. This can be attributed to the electrostatic attraction between the POM anions and Co^2+^ cations throughout the crystal growth^30^. Also, it could be ascribed to the sensitivity of ZIF-67 towards the acidity of the Polyoxometalates (PMA and PTA)^31^. In particular, the PTA@ZIF-67 sample has demonstrated a least uniform structure due to the higher acidity of PTA in comparison with PMA^32^. Moreover, the presence of W and P from the PTA in the PTA@ZIF-67 sample and Mo and P from the PMA in the PMA@ZIF-67 sample respectively along with Co, N and C from ZIF-67 was confirmed using the EDX spectrum of the fabricated POM@ZIF-67 hybrids, see Fig. 2d–f.

**Figure 3:**
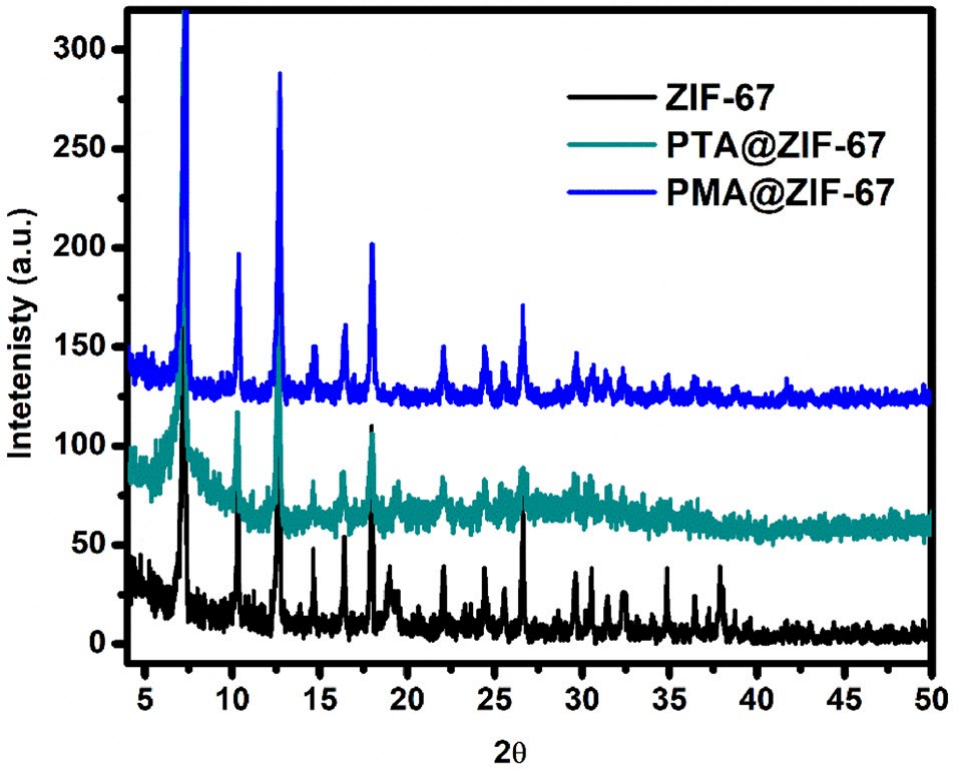
Shows the XRD patterns of before and after encapsulated ZIF-67 samples.

X-Ray Diffraction is a widely used tool for the investigation of materials diffraction peaks and their crystallinity as shown in Fig. 3. The XRD pattern of the bare MOF demonstrates diffraction peaks at 2θ = 7.23°, 10.34°, 12.65°, 14.7°, 16.61°, 17.95°, 22.7°, 24.55°, 25.6°, 26.67°, 29.52°, 31.36°, 32.37° which are ascribed to (011), (002), (112), (022), (013), (222), (114), (233), (224), (134), (044), (244) and (235) reflections. The XRD measurements verified the construction of pure phase of the parent ZIF-67 MOF material as the diffraction peaks coincide perfectly with the previous reports^16,33^. Moreover, upon the encapsulation of the POMs within the MOF cavities, the XRD patterns revealed that the POM@ZIF-67 composites have conserved the original crystal structure of the parent ZIF-67. Additionally, no further diffraction peaks of bulk PTA or PMA were distinguished in the POM@ZIF-67 composites. This indicates the homogenous distribution of the amorphous POMs Keggin units within the surface of ZIF-67^16^.

**Figure 4:**
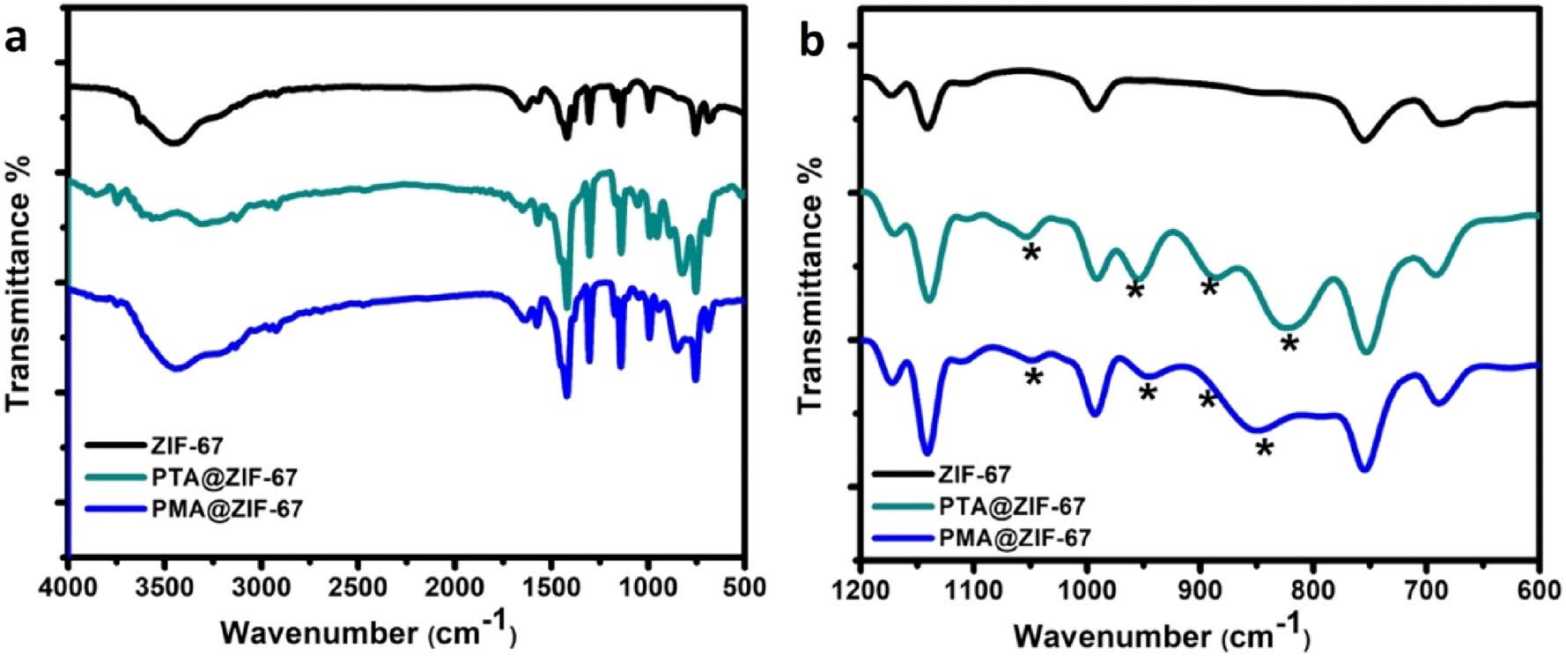
FTIR for pure ZIF-67, PTA@ZIF-67, and PMA@ZIF-67 MOF materials.

FT-IR spectra of ZIF-67 sample and POM@ZIF-67 composites are shown in Fig. 4a. The FTIR spectrum of the parent ZIF-67 MOF shows a signal at 424 cm^−1^ related to the stretching vibration of the Co–N due to the coordination between Co and the N atom of the 2-MIM linker^34^. While the two absorption bands at 690 and 754 cm^−1^ are ascribed to the out of plane bending of the imidazole ring^35^. Also, the bands at 992 and 1141 cm^−1^ demonstrate the bending and stretching vibrations of the C–N bond in the aromatic 1,3-diazole ring, respectively^36^. Furthermore, the stretching vibration of the C=N is identified from the absorption band at 1572 cm^−1^ while the bending vibration of the N–H bond is verified from the peak at 1640 cm^−1^ ^35^. Besides, the two bands detected at 2923 and 3131 cm^−1^ are ascribed to the aliphatic and aromatic C-H stretch of 1,3- diazole, respectively^35^. Whereas, the broad peak located between 3200 and 3700 cm^−1^ is ascribed to the vibration of O–H in the bonded water^37^. On the other side, the FTIR spectra of POMs@MOF composites reveals the appearance of new four vibrational bands located at 1050, 945, 850, and 778 cm^−1^ as indicated in Fig. 4b. Those vibrational bands are characteristic of the Keggin-type POM units. The absorption bands at 778 and 850 cm^−1^ are assigned to the M–O–M edge sharing bond and M–O–M vertex bond in the POM units, respectively where (M = Mo or W)^38^. While the bands at 945 and 1050 cm^−1^ are attributed to the terminal M = O bond and the asymmetrical stretching vibration of the P–O in the central PO_4_ tetrahedron, respectively^38^. Thus, the successful encapsulation of POMs anions within the MOF cavities without altering their structure in the final composite material is confirmed by depicting the characteristic vibrational peaks of both POMs (PMA or PTA) and ZIF-67 in the FTIR spectra of POMs@MOF composites.

**Figure 5:**
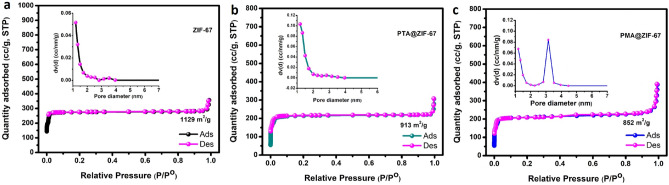
N_2_ adsorption–desorption isotherms of the fabricated (**a**) bare ZIF-67, (**b**) PTA@ZIF-67 and (**c**) PMA@ZIF-67 samples with the insets showing the corresponding pore size distribution.

The N_2_ adsorption–desorption measurements were conducted to investigate the textural properties of the fabricated ZIF-67 and POM@ZIF-67 materials, Fig. 5. The N_2_ adsorption–desorption isotherm of the bare MOF demonstrates a large N_2_ uptake at low relative pressures indicating the characteristic type I isotherm which is typical for microporous materials. As noted from Fig. 5, the POM@ZIF-67 composites have conserved the microporous texture of the parent MOF demonstrating a type I isotherm as well but with less amount of N_2_ uptake which can be ascribed to the partial incorporation of the POM units within the cavities of ZIF-67. The ZIF-67 sample has demonstrated a high specific surface area (SSA) of 1129 m^2^ g^−1^ and a total pore volume of 0.569 cm^3^ g^−1^. As expected, the POM@ZIF-67 composites exhibited a lower SSA and total pore volume compared to that of the bare ZIF-67 sample, where PTA@ZIF-67 sample displayed a higher BET SSA of 913 m^2^ g^−1^ compared to that of the PMA@ZIF-67 (852 m^2^ g^−1^). The N_2_ adsorption–desorption measurements confirmed the successful encapsulation of the Keggin POM units inside the pores of ZIF-67, in agreement with the FTIR results. It is remarked that the prepared composite materials still demonstrate a relatively high pore volume and SSA, which are critical influences in the antibacterial activity.

### Antibacterial activities of ZIF-67 and ZIF-67@POMs

**Figure 6:**
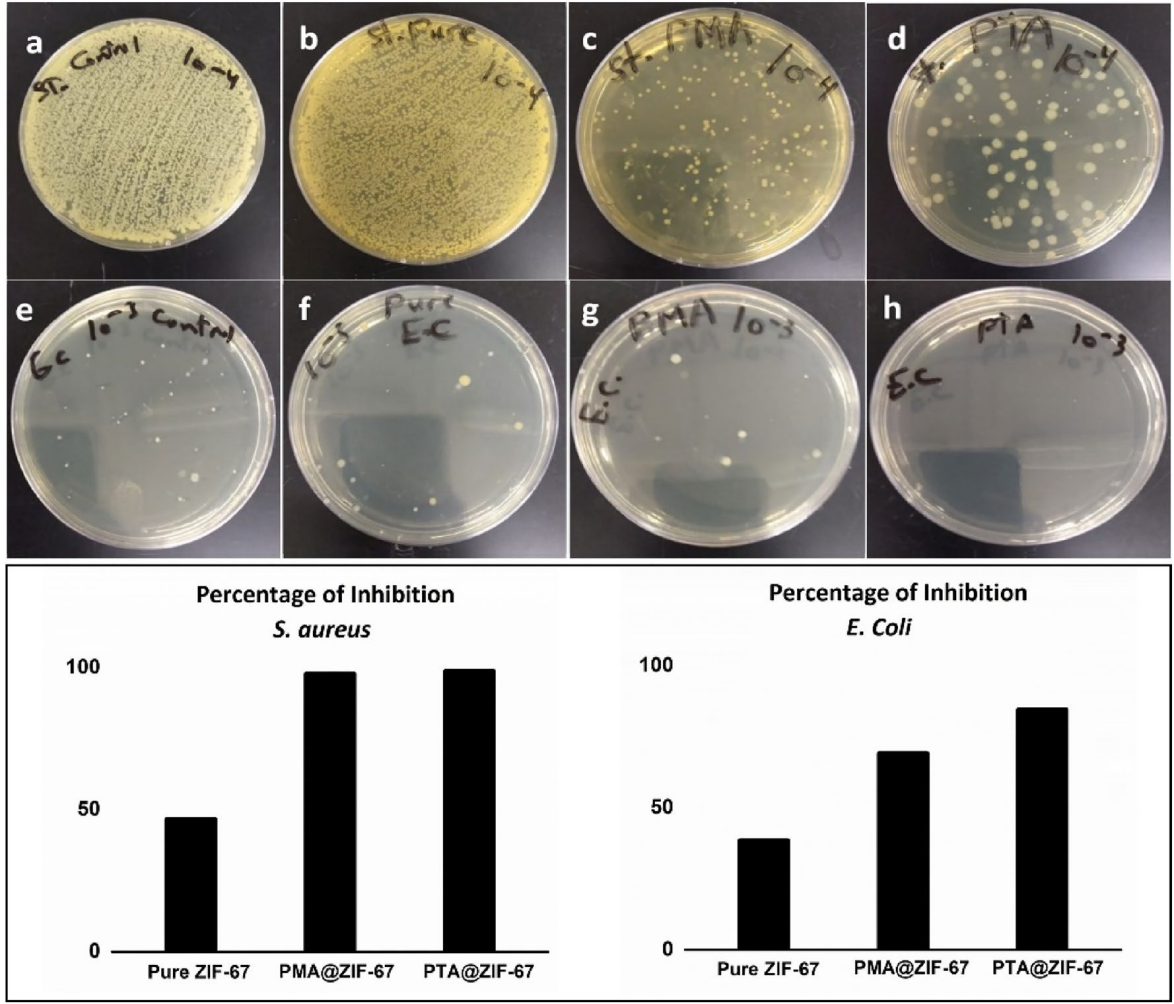
*Gram-positive S. aurous* bacterial strain against (**a**) *S. aurous* Control (**b**) Pure ZIF-67 MOF (**c**) PMA@ZIF-67 (**d**) PTA@ZIF-67 and Gram-negative *E. Coli bacterial* strain against (**e**) *E. Coli* Control, (**f**) Pure ZIF-67 MOF, (**g**) PMA@ZIF-67, (**h**) PTA@ZIF-67.

MOF-based nanomaterials have recently received significant attention as they are used to combat pathogenic bacteria^39^. In our study, the assessment of the ZIF-67 MOF nanoparticulates encapsulated POM against bacterial activity, a colony forming unit (CFU), bacterial counting test was implemented. The ZIF-67 MOF nanomaterials encapsulating both types of Keggin POMs composite showed remarkable antibacterial agents. They experimented against both *E. coli* and *S. aureus*, as a model of pathogenic Gram-negative and Gram-positive bacteria respectively. As indicated in Fig. 6, the ZIF-67 encapsulated POMs MOF possesses unique morphologies on the nanoscale and gives better results compared to the pure ZIF-67. They exhibited a dual effect when POMs incorporated into ZIF-67 MOF nanomaterials revealing a bacterial inhibition growth by 84.6% for *E. coli* and 98.8% for *S. aureus* in PTA incorporated in ZIF-67 MOF nanoparticulates and 69.2% for *E. coli* and 97.8% for *S. aureus* in case of PMA compared to the control sample. PTA@ZIF-67 showed higher bacterial inactivation against both bacterial species rather than the PMA@ZIF-67. The experiment was done in dark conditions^40^.

Several mechanisms have been reported to demonstrate the antibacterial mode of action of nanomaterials including chemical mechanisms, which depend on the generation of reactive oxygen species (ROS) and oxidative stress at the bacterial cell membrane^41^. Moreover, physical and mechanistic antibacterial mechanisms through the close contact of MOF sharp edges with the bacterial membranes cause stresses to the cell membrane and consequently loss of bacterial membrane integrity^41,42^. The MOF nanomaterials exhibited exceptional antibacterial effect, particularly ZIF-67^43^. This is attributed to their porous structure and their ability to function as a source of metal ions^13,44^. Interestingly, Zheng et al. have attributed the antibacterial activity of ZIF-67 MOF to a chemical interaction mechanism through the ROS and found that ZIF-67 has a promising antibacterial activity against Gram-negative bacteria *E.coli* in destroying the bacterial membrane solutions^13^. The Co^2+^ ions that are present in the ZIF-67 MOF NPs have the ability to cause cell wall rupture by generating ROS mediated toxicity^13^. This structural changes and deformation in the bacterial cell wall lead to damages in the intracellular components, proteins, phosphate amine and fatty acid groups of the peptidoglycan in the bacterial cell wall^41,44^. Instantaneously, a complete breakdown of the glycan backbone and the phospholipid in addition to a DNA damage upon the exposure of the nanoparticles to the nucleus^40^. As a result of the generated ROS throughout the leaching process and due to the interaction of ZIF-67 MOF nanoparticulates with biological moieties of the bacterial cell wall, they initiating adverse effects on the cell wall constituents, and leading to cell rupture along with various other extracellular components disruption^40,44^. These trigger events make the ZIF-67 has a potential antibacterial agent. This is in agreement with Kalati et al*.* who studied the effect of ZIF-8 MOF nanostructured as antibacterial agents, highlighting the harmful effect of different morphologies of nanomaterials against Gram-negative and Gram-positive bacteria for metal ion release for bacterial inactivation^44^.

Significant attention has been paid to the use of POMs in the field of antimicrobial water disinfection. They were utilized for destroying microbes and bacteria from water, which is attributed to its high water solubility^11,45^. They possess superior antimicrobial activity, stability, and biocompatibility, which have qualified them to be an excellent material for water microbial purification. Additionally, the incorporation of POMs materials into Nano-sized MOFs may increase their active site numbers and enhance materials surface properties of materials^46^. There are numerous aspects that contributed to triggering biochemical reactions and leading to deleterious effects and serious chemical/biomolecular transformations to bacterial cell wall and growth inhibition. The crystalline phase of MOF materials, sharp edges structure of nanoparticulates of the composite, and a high surface area could be factors for qualifying them to be outstanding antibacterial agents^42^. Herein, in our study, we used the same concentration to test the composites of POM@ZIF-67 MOF nanomaterials and pure ZIF-67 MOF materials against bacteria for water microbial purification. As observed from the FESEM images, there are sharp edges of the ZIF-67 encapsulated POMs that may result in damaging the cell wall of both types of bacteria. The high membrane destruction was observed in *S. aureus*, Gram-positive bacteria and this matched with Jovanovic et al*.* Most importantly, the size of MOF nanoparticulates composite was contributed to the cell rupture by small sizes of NPs in phospholipid layers. Consequently, the inactivation of the bacterial growth was ended by a cell wrapping mechanism that was explained by Perreault et al*.*^47^ Similarly, surface area has an influence on the materials as observed in Fig. 5.

**Figure 7:**
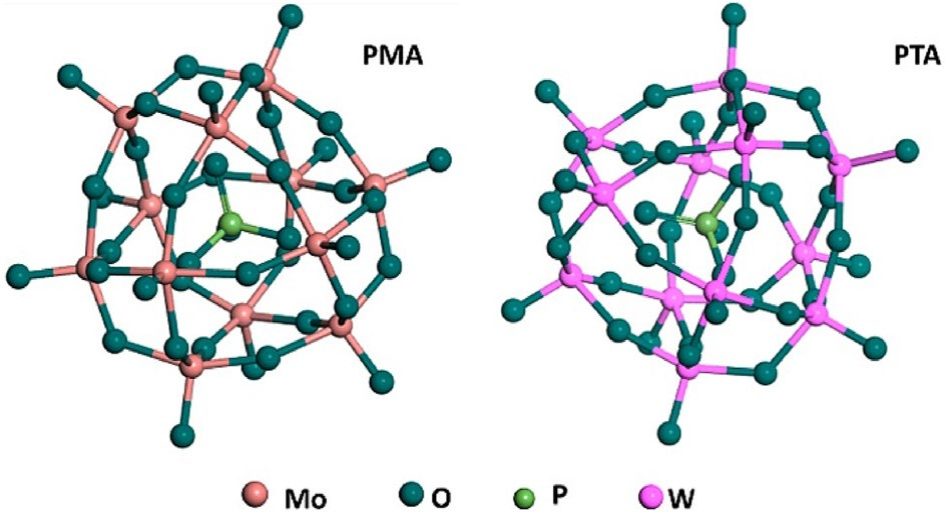
Optimized structures for PMA and PTA.

**Figure 8:**
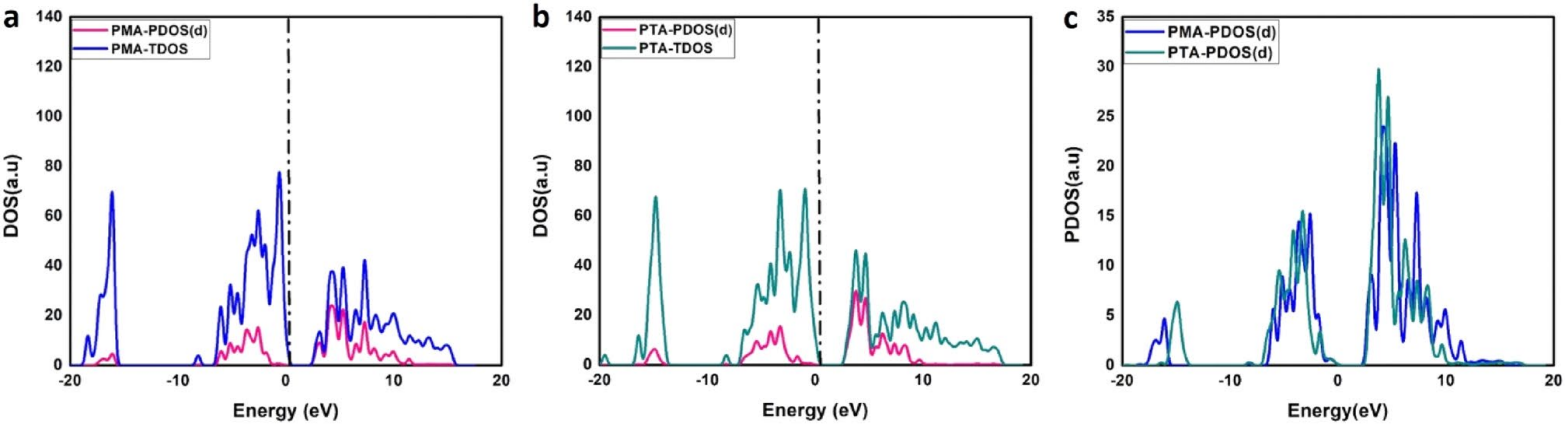
Total density of states (TDOS) and projected density of states (PDOS). (**a**) PMA, (**b**) PTA, and (**c**) PDOS of PTA and PMA.

By DFT calculations the structures for PMA and PTA were optimized as cleared in Fig. 7. It is observed that PMA has wider band gap energy than PTA as shown in Fig. 8. PTA and PMA have band gap energies of 2.81 and 2.98 eV, respectively, which are very close to the reported experimental values of 2.82 and 3.0 eV^48,49^. For PTA, the electrons are localized at the conduction band edge as it is denser and sharper than PMA as depicted in Fig. 8 right side. The lower energy band gap of PTA means better electrical conductivity and more release of electrons which is necessary for initiating cell death^50^. These electrons react with bacteria cell or the liquid medium. Transfer of electrons within the energy states causes excitation of electrons, yielding electron–hole pairs that form reactive oxygen species (ROS). Those ROS facilitate the permeability of the cell, which destroy the bacterial cells by devastating their DNA, proteins, and cell membranes^51,52^. This may be the reason for the higher antimicrobial activity for PTA than PMA.

## Conclusion

In this paper, we introduced MOF nanomaterials incorporated POMs such as PTA and PMA bioactive agents as a dual antibacterial agent for water microbial purification. The materials were prepared through a facile one step co-precipitation synthesis reaction. The structural and surface characterization techniques such as EDX, FTIR, XRD and Surface area measurements confirmed the successful incorporation of the Keggin POMs units within the MOF cavities without any destruction in the framework. The antibacterial activity test using (CFU) revealed that the PTA@ZIF-67 composite demonstrated the highest bacterial inhibition growth by 84.6% for *E. coli* and 98.8% for *S. aureus* compared to that of the PMA@ZIF-67 composite (69.2% for *E. coli* and 97.8% for *S. aureus*). The enhanced antibacterial activity of PTA@ZIF-67 over that of PMA@ZIF-67 was interpreted by DFT calculations which unveil their electronic structure and accounted this for the smaller band gap energy for PTA than PMA. Moreover, it was also ascribed to the higher SSA exhibited by PTA@ZIF-67 which offered more active antibacterial spots.

## Data Availability

The datasets used and/or analysed during the current study available from the corresponding author on reasonable request.
